# Initiating Prognostic Talk During Hospice Multidisciplinary Team Meetings: A Conversation Analytic Study

**DOI:** 10.1177/08258597241286347

**Published:** 2024-11-14

**Authors:** Andrea Bruun, Nicola White, Linda Oostendorp, Patrick Stone, Steven Bloch

**Affiliations:** 1 Marie Curie Palliative Care Research Department, Division of Psychiatry, 4919 University College London, London, UK; 2 Department of Public Health, Children's, Learning Disability and Mental Health Nursing, Kingston University London, London, UK; 3 Department of Language and Cognition, Division of Psychology and Language Sciences, 4919 University College London, London, UK

**Keywords:** prognosis, multidisciplinary care team, hospice, group meeting, communication

## Abstract

**Objective:** Guidelines recommend that patients’ prognoses should be discussed by the palliative care multidisciplinary team. However, there is a lack of evidence on how multidisciplinary teams carry out prognostic discussions, and especially how prognostic talk is initiated during team meetings. This study explored how prognostic talk is initiated and responded to during meetings of a hospice multidisciplinary team. **Methods:** Video-recordings of 24 inpatient multidisciplinary team meetings in a UK hospice were collected from May to December 2021. A total of 65 multidisciplinary team members participated in the meetings. Recordings were transcribed and analysed using Conversation Analysis. **Results:** Prognostic talk was initiated during multidisciplinary team members’ patient case presentations. Case presentations followed a certain template, and prognoses could be initiated as responses to template items such as the patient's Phase of Illness and Karnofsky's Performance Status score and the patient's main diagnosis and issues. Prognoses also occurred as accounts for a lack of template item responses. Beyond the patient case presentation, prognostic talk was initiated in relation to discharge planning. Prognoses appeared with sequences of assessments that accounted for them. When a prognosis was provided, it received confirming minimal responses from other team members. **Conclusions:** Patients’ prognoses were embedded into other care discussions during meetings of a hospice multidisciplinary team. These findings can be used to inform the development of clinical guidelines and interventions aiming at improving multidisciplinary team discussions around prognosis in the future.

## Introduction

Multidisciplinary teams are essential for providing holistic palliative care,^1^ and teams should ideally meet weekly to review patients’ care plans.^2^ Recommendations state that the multidisciplinary team should be consulted to determine the prognoses of palliative care patients.^3–6^ Prognoses are important as clinical decision-making regarding discharge planning, cardio-pulmonary resuscitation, goals of care, and enrolment onto integrated care pathways rely on them.^7^ However, there is a paucity of evidence on how multidisciplinary teams discuss patients’ prognosis.^8–10^

Studies have addressed how professionals communicate prognoses to patients and their next-of-kin.^11–13^ Findings show how different communicative strategies are employed to navigate prognostic discussions with patients,^14,15^ with several guidelines on delivering them.^16–18^ Research has been conducted specifically on how such discussions are initiated.^15,19,20^ However, there is no guidance on how multidisciplinary teams themselves should engage in prognostic discussions and particularly how hospice multidisciplinary team members initiate prognostic talk during their meetings. This study aims to explore how prognostic talk is initiated and responded to during meetings of a hospice multidisciplinary team.

## Methods

This was an applied Conversation Analysis (CA) study involving collection and analysis of video-recorded hospice multidisciplinary team meetings. CA is a research approach used to systematically analyse social interaction through close investigation of how participants produce turns at talk.^21^ Methodological tools include recordings of interactions and detailed transcriptions of these data. Analyses involve describing the interactional structure in terms of how practices, actions, and activities are organised by and between speakers. Thus, a CA approach allows for detailed exploration of *how* prognostic talk is carried out (ie, initiated and responded to) by staff members during these meetings.

The study protocol was registered with the Open Science Framework (OSF) on 04 June 2021 (https://osf.io/bdf3t). It was part of a wider project exploring prognostic decision-making of imminently dying patients within specialist palliative care multidisciplinary teams.^22^

### Study Setting

Data were collected from a UK hospice providing services for patients with advanced life-limiting diseases. The hospice comprised day care, outpatient facilities, and an inpatient unit with two 15-bed wards. A weekly one-hour ward multidisciplinary team meeting was held to discuss patient care.

### Study Participants

Participants were hospice staff (eg, doctors, nurses, physiotherapists, and social workers) and visitors attending the inpatient multidisciplinary team meeting. Any meeting attendee who was willing to provide informed consent was eligible for the study. Patients did not attend these meetings.

### Participant Consent

Written consent was obtained from 65 meeting attendees. If a staff member did not consent to participate in the study, the meeting was still recorded but their data were not analysed.

### Data Collection

Data were collected from May to December 2021. During this period, only one ward was operating due to COVID-19 restrictions. Two cameras and an audio-recorder were used for data collection, and one researcher [AB] was present as an observer.

### Data

The final dataset comprised video-recordings of 24 meetings (approximately 24 h of data), with 10-15 attendees each.

### Data Management and Analysis

Recordings were audibly masked by removing all participant identifying information (ie, patient and staff names and locations). Sequences involving prognosis were identified and transcribed following standard CA conventions (see Supplementary File 1).^23,24^ CLAN software version 2021-04-28 or above was used for the transcription process. Single-case analyses^25^ were conducted to create a collection of cases (ie, collection analysis^26^) systematically exploring patterns of prognostication in the interactions. Data and analyses were discussed in data sessions; a common practice within CA research.^27^

In this paper, prognostic talk is defined as utterances conveying when patients are expected to die. A previous paper described how prognostication was carried out in different ways during hospice multidisciplinary team meetings and included stating the patient's current health status or using unspecific and specific time period references.^28^

## Results

The hospice multidisciplinary team initiated prognostic talk through completing meeting template information during patient case presentations and through discharge planning considerations where the patient's prognosis was important.

### Patient Case Presentations

Staff initiated prognostic talk during patient case presentations where nurses and healthcare assistants presented the patients they had overseen, using a template. The template included:

- Name and age
- Phase of Illness
- Diagnosis and main issues
- Action points from the last multidisciplinary team meeting
- ‘What Matters to Me’

The nurse and healthcare assistant would fill in their response to each template item for each patient before the meeting. The meeting template items and the responses were then shared with the team by the presenter reading them aloud. Patients’ prognoses were included within item responses presented by staff. It was often simply stated that the patient was dying, and staff would move on to discuss any final plans for the patient.

Case presentations involved a structure where the presenter had the interactional floor and produced an extended turn-at-talk, suspending the usual turn-by-turn talk. Other team members aligned by supporting the structural asymmetry of the case presentation activity: that the presenter had the floor until their presentation was complete. During case presentations, other team members remained silent or only provided minimal responses through continuers such as *hm, yeah*, and nods that treated the presenter's turn as still in progress.^29^ The presenter would read aloud the meeting template items and their responses to them based on their time with the patient and the care provided during the day. In this way, they also engaged in a reported text activity.^30^ They might switch between formulations that clearly reflected written formulations between more non-text-based utterances.

### Response to “Phase of Illness and Karnofsky Performance Status Scale” Meeting Template Item

The dataset revealed several cases where mentions of patients’ prognoses were initiated through the response to the patient's Phase of Illness. Phase of Illness is a tool used in advanced illness to describe distinct stages of an individual's illness according to their care needs.^31^ There are four phases: *Stable*, *unstable*, *deteriorating*, and *dying*. When having to assess the patient's Phase of Illness, prognosis is an inevitable consideration – especially for the last two phases. Excerpt 1a shows how the patient's Phase of Illness conveys prognostic information. The nurse (NUR) begins the presentation of the next patient for multidisciplinary team discussion.


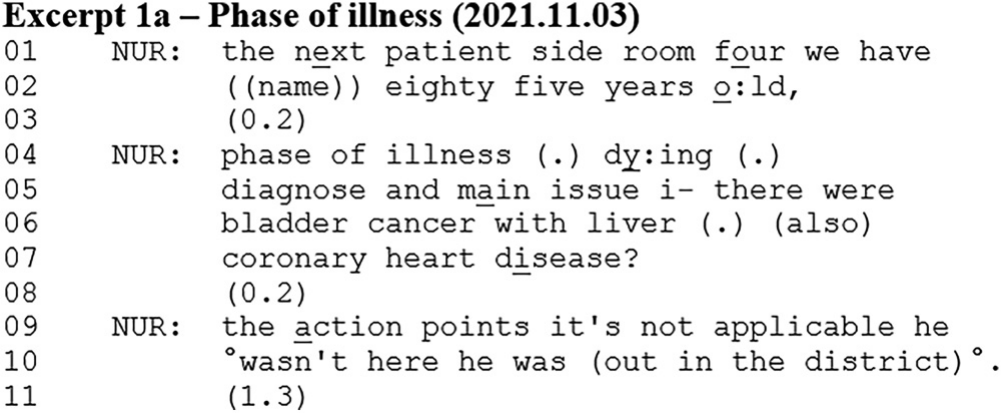


The nurse follows the meeting template agenda and begins with basic patient information (ie, hospice room, name, and age). She continues with the patient's Phase of Illness item, which is recorded as *dying* (line 04)*.* This utterance describes the patient's prognosis and is initiated by the nurse having assessed his Phase of Illness. She then lists the patient's diagnosis and main issues, and the lack of action points from last week's meeting.

In this excerpt, the prognostic talk occurred during the nurse's patient case presentation. The nurse reported what she had written down as her response to the presentation template item about the patient's Phase of Illness. This meant that the patient's prognosis was part of, or embedded into, the nurse's patient case presentation, which involved reporting her responses to the meeting template, during which she had the interactional floor. Aligning with the extended turn-at-talk structure, the nurse did not receive responses that either confirmed, rejected, or questioned the prognosis during her presentation. The patient's Phase of Illness was often followed by the patient's Karnofsky Performance Status (KPS)^32^ score, as these two were listed together on the hospice handover sheet.

### Accounting for Lack of Response to Meeting Template Items

Examining the rest of the patient case presentation from Excerpt 1a, another prognosis occurred shortly after the one already presented.


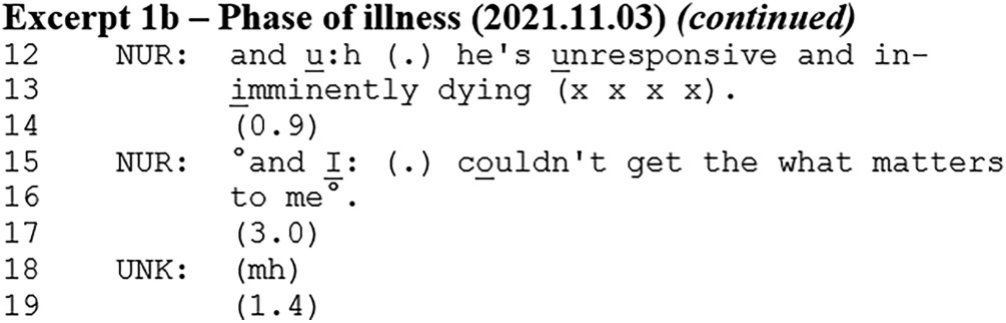


The nurse states that the patient is unresponsive, followed by a prognostic formulation (line 13). The nurse says that the patient is *imminently dying*. After a pause, she states that she could not obtain the ‘What Matters to Me’ from the patient. In this way, the statement of unresponsiveness and the prognosis of imminently dying account for the lack of a ‘What Matters to Me’ record. Aligning with the extended turn-at-talk, this does not elicit a response from other team members. After this excerpt, the ward manager mentions that a colleague has spoken to the patient's daughter, and they depart from the prognostic talk. The patient's prognosis is not discussed further.

This analysis shows how the prognosis was used to account for not having obtained the ‘What Matters to Me’ meeting template item. However, this prognosis was the nurse's *own* assessment of the patient going beyond the response to the Phase of Illness item presented in Excerpt 1a. That this is the nurse's own upgraded prognostic assessment is seen through the word *imminently* that goes beyond the five Phases of Illness (ie, *stable, unstable, deteriorating*, and *dying*).

The prognosis in Excerpt 1b was part of the patient presentation, where the nurse had the interactional floor and other members aligned by remaining silent, similar to Excerpt 1a. Interestingly, the prognosis did not receive a response *after* the patient presentation, where the floor was open for other team members.

### Response to “Main Diagnosis and Main Issues” Meeting Template Item

A prognosis also occurred as a “result” of the patient's diagnosis and main issues item of the case presentation format. In Excerpt 2, a nurse (NUR) is presenting different symptoms, assessments, and interventions, which leads her to provide an upgraded prognosis for the patient. Just before the Excerpt, the nurse introduced the patient and her ‘What Matters to Me’ record.


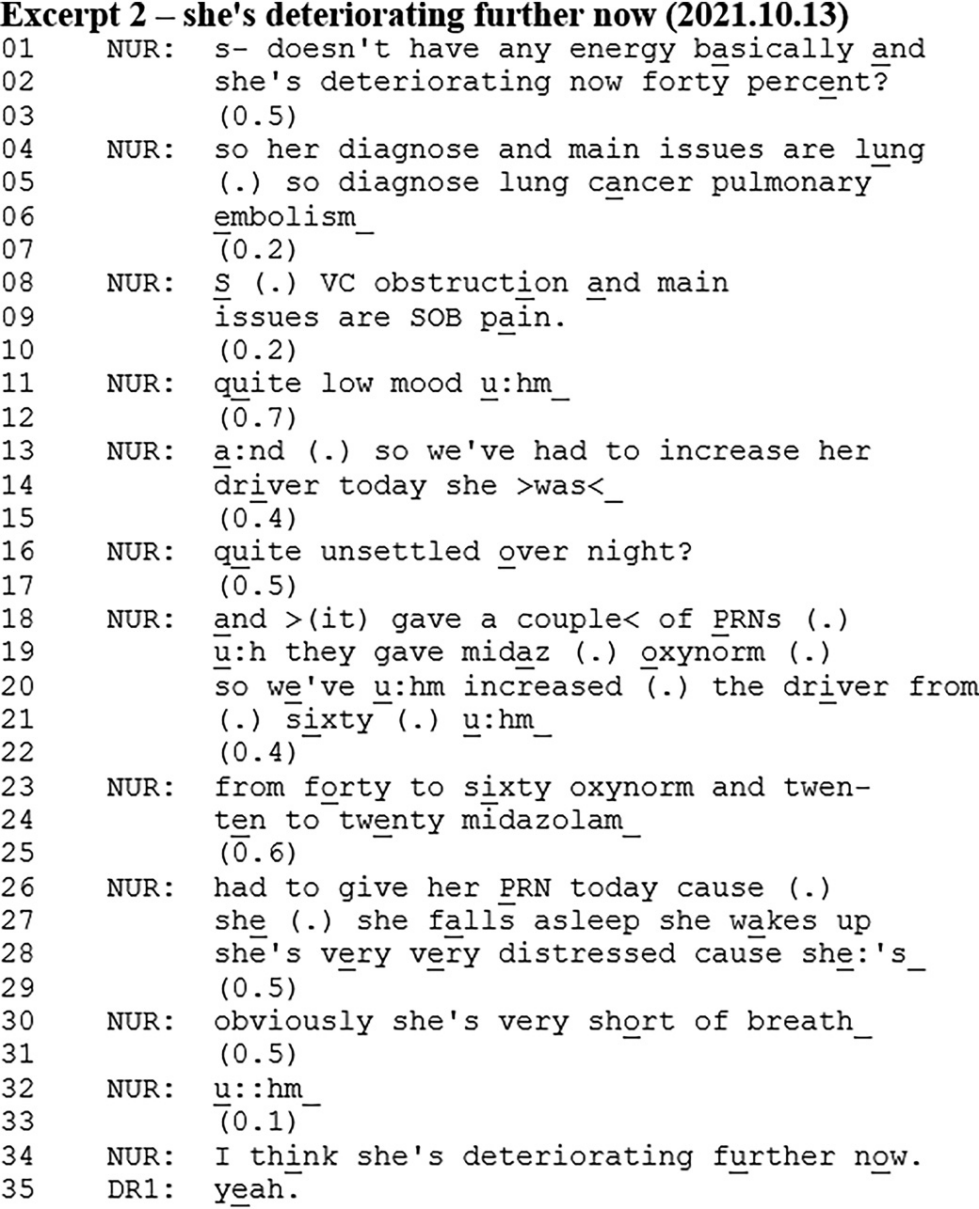


The nurse presents the patient's Phase of Illness and KPS score, diagnosis, and then she continues with the main issues. The presentation of the main issues involves the unfolding of a sequence where the nurse presents multiple symptoms, assessments, and interventions (lines 09-30). The sequence ends with the prognosis (line 34), where the nurse states that she thinks the patient is *deteriorating further now*. In this way, the sequence comprising the nurse's assessments accounts for the prognosis. The prognosis also has a summarising character, where it seems to conclude or be a product of the nurse's assessment of the patient. In this way, the sequence leads to an *upgraded* prognosis compared to the prognostic utterance provided by the nurse earlier (ie, *deteriorating now at 40%* in line 02). Lastly, the prognosis receives an affiliative response from the doctor (DR1) that displays agreement with the nurse.

This prognosis occurred as a conclusion or result of the nurse's sequence assessing the patient when listing the patient's main issues. This sequence accounted for the prognosis and provided evidence for why the nurse arrived at this prognosis. The prognosis was then justified and grounded within the sequence comprising the nurse's assessment of the patient. It was also seen how this sequence led to an upgraded prognosis.

What these three different ways of initiating prognostic talk have in common is that they receive either no response or minimal responses simply confirming or accepting the prognosis, which is not treated as being noticeable nor problematic by the participants in the interaction.

### Discharge Planning

Questions about discharge also initiated prognostic talk. Patients’ prognoses were crucial to consider in relation to whether the patient was suitable for discharge. Such discussions usually involved consideration of the options for either discharging the patient to their own home or to a nursing home. If neither of these were appropriate due to the patient approaching death, then a third option would be for them to stay in the hospice. In these cases, it appeared uncertain whether the prognosis was “poor enough” for the patient to stay in the hospice or whether they should be managed elsewhere.

Before Excerpt 3, the nurse (NUR) has just completed the patient presentation and listed the patient's action points from the last meeting. One of the action points was discharge planning.


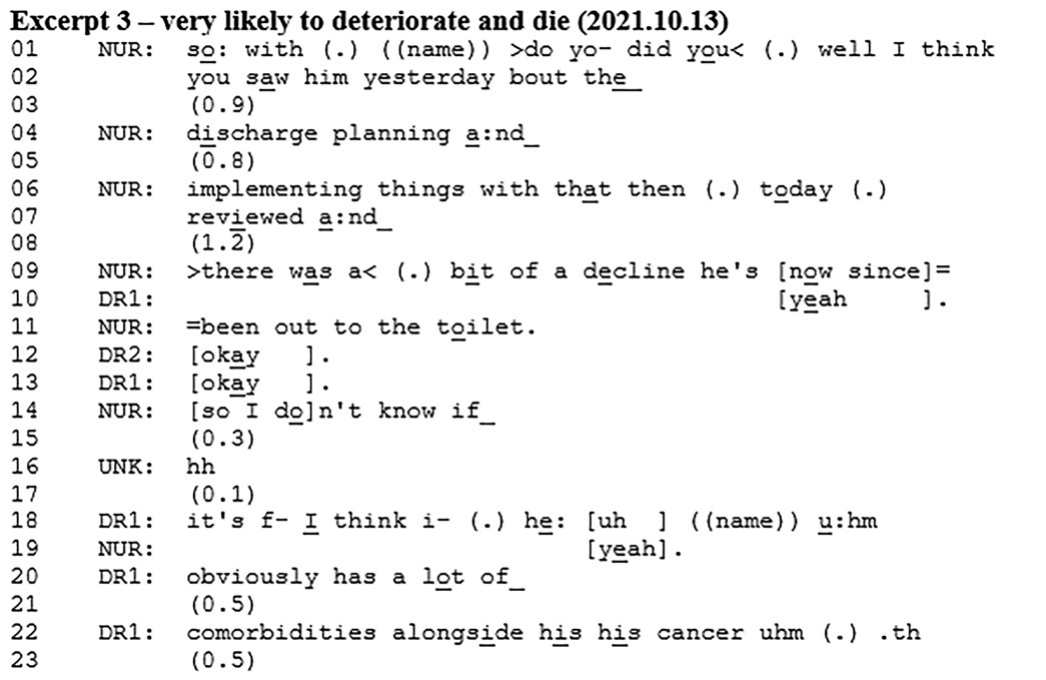
 
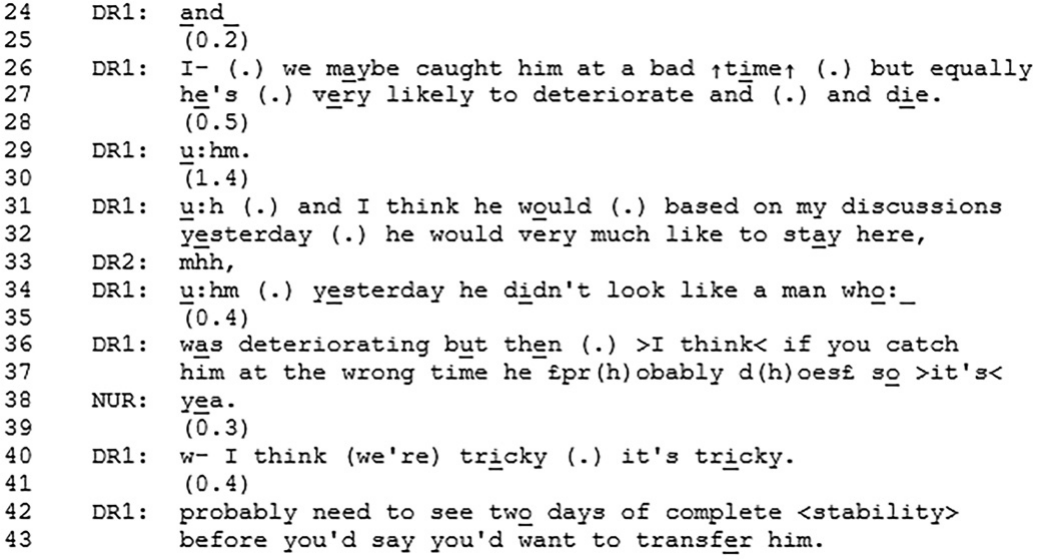


The nurse talks to the doctor (DR1) about the doctor having seen the patient regarding discharge planning. These utterances seek confirmation from the doctor as they involve the doctor's work with the patient (ie, A-statements about B-events^33^). The nurse presents a fluctuating picture of the patient's state in which the team was planning on discharging the patient yesterday, but then the day after the patient deteriorated. However, other evidence indicates that the patient is instead doing better. The nurse ends the description of the patient's unstable state with, *so I don’t know if* (line 14) displaying a degree of uncertainty regarding the discharge plan. The doctor states that the patient has several comorbidities that make him unstable, which seems to account for the provided prognosis (lines 26-27). The doctor stresses the uncertainty of the patient's state by saying that this might be a current (bad) time for the patient but also that this could be a step in the trajectory where the patient is very likely to deteriorate and die. The prognosis is followed by a statement of the patient's own wish to stay in the hospice. The doctor then provides assessments of the patient that orients towards the difficulty of their fluctuating state and prognosis (lines 34-40). She then states that the condition for discharge planning is that the patient should be stable for two days. After the Excerpt, the discussion continues about discharge plans during which other prognoses and statements also occur. After a while, the team agrees on postponing the discharge plans for the following week.

The nurse's utterances at the beginning of the excerpt display that decline impacts discharge plans – and that improvement (or stability) does as well. Instead of providing a prognosis herself, she provides an opportunity for the next speaker to elaborate on the decline and to potentially provide a prognosis. The doctor is expected to respond since she has discussed discharge plans with the patient. Instead of providing a straightforward answer to the discharge issue, the doctor then provides an account and a prognostic utterance. By providing the prognosis at this point in the interaction, it appears relevant or even conditional to consider the patient's prognosis when deciding on discharge plans. The doctor aligns with the nurse's invitation to elaborate on the decline, which leads the doctor to provide the prognosis. In this way, there is orientation from both the nurse and the doctor towards decline and prognosis as conditional to discharge plans. It is noteworthy that the prognosis does not receive a verbal response despite the long pause following it, which provides a clear opportunity for other team members to respond. This might lead the doctor to continue speaking. It should, however, be mentioned that in the recording, there is no visual access to the nurse so she could be confirming non-verbally.

Excerpt 3 illustrated how discharge plans were dependent on the patient's clinical state and therefore prognosis. There was an orientation from both the nurse and the doctor towards prognosis as being conditional to discharge planning. The prognosis was provided by the doctor in response to the nurse's statement about the doctor's work with the patient.

## Discussion

### Main Findings of the Study

Prognoses were embedded into patient case presentations or through discharge planning discussions during meetings of a hospice multidisciplinary team. During case presentations, prognoses appeared as responses to presentation items such as the Phase of Illness and KPS score. A prognosis could also account for a lack of response to other presentation items. Prognoses occurred as a result of a sequence with assessments of the patient as part of the diagnosis and main issues item. Prognoses also appeared when they were necessary to consider regarding patient discharge plans. They appeared with sequences with assessments of the patient that accounted for them. Prognoses received confirming minimal responses from other members and were responded to in a manner that did not directly challenge or further negotiate them.

### What This Study Adds

That prognostication is not an isolated event during meetings of a hospice multidisciplinary team aligns with research showing how prognoses are discussed in relation to other care aspects.^8^ Staff have also explained how they make discharge plans based on patients’ prognoses.^34^ The prognostic decision-making process is not a static event but fluid because the distinction between active patient management and dying is blurred.^35^ This study confirms this by showing that prognoses occur with sequences comprising assessments of the patient. When certain patient assessments (eg, the patient is no longer eating and drinking, lacks mobility and is sleeping a lot) are listed, it might imply that the patient is now close to death. Necessary care decisions are then made that might involve prognostication by reacting to certain prognostic factors.

When prognoses were embedded in the case presentations, responses were minimal or absent. This might be due to the presenter's extended turn-at-talk, where recipients should “give” them the interactional floor. In this way, the patient presentation, and any embedded prognosis, does not invite input or ratification from team members. However, it has been shown that nodding, when provided mid-telling, conveys preliminary affiliation with the teller's position.^29^ A detailed analysis of head movements has not been conducted, but staff members frequently nodded and thereby displayed agreement. Future research should explore this in more detail. Meeting interactions may also be special in the sense that formulations that are not responded to are treated as being accepted, whereas group silence is the preferred and sufficient response.^36^

As noted previously, clinical guidelines and recommendations do not specify *how* prognoses should be discussed by the multidisciplinary team.^3–6^ The National Institute for Health and Care Excellence simply recommend to “discuss the dying person's prognosis with other members of the multi-professional care team”,^5^ while another recommendation states that, when possible, a discussion with a multi-professional team should be conducted as this may help refine prognostic estimates.^3^ This may imply that the team should somehow negotiate patients’ prognoses, and that the team should engage in discussions with the aim to accurately predict and/or refine a prognosis. However, this study showed that prognostic talk received minimal confirming responses. It is important to note that such responses were not treated as noticeable nor problematic by participants themselves in the interaction. These minimal responses were sufficient for the job at hand. In this way, in-depth prognostic discussions into accurately predicting or refining patients’ prognoses were not deemed a priority nor a necessity by the meeting participants.

That prognosis is embedded within other care aspects further supports the claim that this team seems to prioritise working towards shared understanding of the patient's case and what is needed (in terms of prognosis) to provide best care.^28^ However, future research should explore how multidisciplinary team members perceive these discussions, and how they would prefer them to happen. Combining the study findings with team members’ views would allow for interventions or guidelines to be developed *with* professionals and not just *for* them.

Future interventions could target how multidisciplinary team members discuss prognoses to see if that had an impact on the ways in which prognoses are negotiated or used in clinical care. One way could be to compare different meeting templates, as these seem to be a key initiator of prognostic talk. The use of a specific proforma collecting prognostic estimates, for example, could help clinicians focus the discussion.^37^

### Study Strengths and Limitations

Data comprised 24 video-recordings of multidisciplinary team meetings; a sufficient number to allow for patterns to occur and to be identified. The use of video-recordings ensured that findings were based on real-life interactions. The detailed analysis of the moment-by-moment interaction provided in-depth insights into how prognostic talk was initiated and responded to. Analyses were discussed in data sessions, ensuring transcription accuracy and validation of findings.

Meetings from only one hospice multidisciplinary team were included in this study. This may therefore be idiosyncratic of this particular team, which challenges the generalisability of the findings. The qualitative nature of the CA approach should be considered where analyses do not aim to be generalisable. However, there is still scope for more research into this field, involving several hospice multidisciplinary teams.

Participants wore face coverings due to COVID-19, which meant that mouth movements could not be used for speaker clarification. Transcription often relied on recognising voices and making reasonable assumptions about who was speaking. Variable sound quality due to background noise also sometimes made it difficult to transcribe participants’ speech.

There was limited visual access to some participants due to the cameras’ range. This meant that it was often not possible to observe non-verbal cues. Non-verbal interaction can be important for social interaction,^38^ and future research should aim to overcome such limitations.

It was not possible to eliminate the possibility of researcher influence on interactions. However, it has been argued that “researcher-participants do not (necessarily) challenge the local ‘naturalness’ of the data”.^39^ The presence of visitors or observers is not regarded as being unusual as they commonly participate in these meetings.

## Conclusion

Patient's prognoses are embedded into case presentations or discharge planning considerations and receive confirming minimal responses during meetings of a hospice multidisciplinary team. Guidelines are vague when providing recommendations about multidisciplinary team prognostication and need to be updated by taking the current evidence into account.

## Supplemental Material

sj-docx-1-pal-10.1177_08258597241286347 - Supplemental material for Initiating Prognostic Talk During Hospice Multidisciplinary Team Meetings: A Conversation Analytic Study Supplemental material, sj-docx-1-pal-10.1177_08258597241286347 for Initiating Prognostic Talk During Hospice Multidisciplinary Team Meetings: A Conversation Analytic Study by Andrea Bruun, Nicola White, Linda Oostendorp, Patrick Stone and Steven Bloch in Journal of Palliative Care
