# Supplementary material for: Initiating Prognostic Talk During Hospice Multidisciplinary Team Meetings: A Conversation Analytic Study
Source: J Palliat Care. 2024 Nov 14;40(2):183–9. doi: 10.1177/08258597241286347 (PMC11967105; doi:10.1177/08258597241286347)
Supplement: sj-docx-1-pal-10.1177_08258597241286347 - Supplemental material for Initiating Prognostic Talk During Hospice Multidisciplinary Team Meetings: A Conversation Analytic Study [file sj-docx-1-pal-10.1177_08258597241286347.docx]

Supplementary File 1 – Transcription Key

| **Symbol/example** | **Explanation** |
| --- | --- |
| [word]  [word] | Overlapping speech |
| (0.2) | Pause in seconds |
| (.) | Micro-pause (under 0.1) seconds |
| word | Stress (more underlining indicates more stress) |
| wo:rd | Prolongation of the preceding sound (number of colons indicates length of prolongation) |
| word=  =word | Latching of speech (no silence between turns/parts of one turn) |
| hhh | Outbreath (number of h indicates length) |
| .hhh | Inbreath (number of h indicates length) |
| WORD | Speech with loud volume |
| °word° | Speech with low volume |
| ↑word  ↓word | Marked pitch change, up (↑) or down (↓) |
| . | Final falling intonation |
| , | Slight rising intonation |
| _ | Level/flat intonation |
| word- | Cut-off of preceding |
| >word< | Speeded up talk |
| <word> | Slowed down talk |
| #word# | Creaky voice |
| £word£ | Smiling voice |
| {word  {gesture | Multimodal element |
| (word)/(x) | Possible hearings/inaudible (x indicates the number of syllables) |
| ((comment)) | Transcriber’s comments |

Adapted from Jefferson (2004) and Hepburn and Bolden (2012).
